# Examining birth preparedness and complication readiness: a systematic review and meta-analysis of pregnant and recently delivered women in India

**DOI:** 10.1186/s12905-024-02932-4

**Published:** 2024-02-14

**Authors:** Tanya Singh, Brajaraj Tripathy, Anuj Kumar Pandey, Diksha Gautam, Sidharth Sekhar Mishra

**Affiliations:** 1 Present Address: Knowledge Management Division, National Health Systems Resource Centre, New Delhi, India; 2 Present Address: Quality and Patient Safety Division, National Health Systems Resource Centre, New Delhi, India; 3 Department of Health Management Research, International Institute of Health Management Research, New Delhi, India; 4 Institute for Population and Social Research, Mahidol University, Nakhornpathom, Thailand

**Keywords:** Birth preparedness, Complication readiness, Pregnant women, Recently delivered women, BPCR

## Abstract

**Background:**

Birth preparedness and complication readiness (BPCR) is an essential component of safe motherhood programs. This study aims to systematically identify and synthesize available evidence on birth preparedness and complication readiness among pregnant and recently delivered women in India.

**Methods:**

The study followed PRISMA guidelines and used databases such as PubMed, Cochrane Library, and ProQuest. Joanna Briggs Institute [JBI] Tool was used for critical appraisal of studies. The meta-analysis was conducted using Comprehensive Meta-Analysis [CMA] tool and R studio software. Statistical heterogeneity was evaluated using visual inspection of the forest plot, Cochran’s Q test, and the I^2^ statistic results. Funnel plot and Egger’s tests were applied to explore the possibility of the publication bias in the studies [PROSPERO: CRD42023396109].

**Result:**

Thirty-five cross-sectional studies reported knowledge on one or more components of birth preparedness [BP], whilst knowledge on complication readiness [CR] or danger signs was reported in 34 included studies. Utilizing the random effect model, the pooled result showed that only about half of the women [49%; 95% CI: 44%, 53%] were aware on BPCR components. This result ranged between 15% [95% CI: 12%, 19%] to 79% [95% CI: 72%, 84%] in Maharashtra and Karnataka respectively [I^2^ = 94%, *p* = < 0.01]. High heterogeneity [> 90%] is observed across all components [*p* < 0.01]. The result of subgroup analysis indicated no significant difference in the proportion on BPCR among pregnant women [50%; 95% CI: 45%, 55%] and recently delivered women [54%; 95% CI: 46%, 62%]. However, the southern region of India indicates relatively better [56%; 95% CI: 45%, 67%] prevalence.

**Conclusion:**

Our study highlights the low prevalence of BPCR in India and the factors associated with it. Scaling up cost-effective interventions like BPCR that have a positive overall effect is necessary. Authors strongly suggests that birth preparedness and complication readiness should be given utmost importance to reduce maternal morbidity and mortality to achieve the Sustainable Development Goals. Consideration should be given to fortifying existing resources, such as frontline workers and primary healthcare, as a strategic approach to augmenting the effectiveness of awareness initiatives.

**Supplementary Information:**

The online version contains supplementary material available at 10.1186/s12905-024-02932-4.

## Introduction

The health of pregnant women, from conception to the postnatal period, must be treated as a priority. Each stage of the process should be a positive experience, enabling both mother and baby to realize their full potential for health and happiness. Globally during 2020, around 800 women per day, or around one every two minutes, died from pregnancy and childbirth related avoidable causes [1]. Nearly 95% of maternal deaths in 2020 occurred in low- and middle-income [LMIC] nations, the majority of which could have been prevented [2]. Of the estimated 253,000 maternal deaths worldwide during 2020, 87% [253,000] occurred in Sub-Saharan Africa and Southern Asia. Maternal mortality ratio [MMR] decreased most substantially in Eastern European countries and South Asian countries between 2000 and 2020, by 70% [from MMR of 38 to 11] and 67% [from MMR of 408 to 134], respectively [1–3].

Approximately three-quarters [73%] of maternal fatalities between 2003 and 2009 are due to direct obstetric causes like severe haemorrhage that typically occur after childbirth, high blood pressure during pregnancy [pre-eclampsia and eclampsia], embolism, unsafe abortion etc. The remaining 27% deaths are due to indirect obstetric causes resulting from previously existing disease like cardiovascular diseases, HIV, severe anaemia, diabetes and hepatitis etc [4, 5]. Despite these known causes, lowering maternal mortality and morbidity has been proven to be significantly impeded by individual pregnant women’s, families’, and medical professionals’ delay in responding to the initiation of labour and the development of complications [6].

Compared to the global average of 43%, India has impressively reduced maternal mortality since 2005 by 77% [7] Between 2017 and 2019 and 2018–2020, MMR in India has significantly reduced i.e., from 103 to 97 deaths per lakh live births respectively, surpassing the target set by the NHP 2017 [8]for 2020. However, to meet the UN’s Sustainable Development Goal of 70 per 100,000 live births by 2030, the MMR must be substantially improved [9, 10]. Unlike other countries, India has also adopted a set of strategies i.e., from single interventions to a complex set of public health intervention for this decline in maternal mortalities [11, 12]. Despite these efforts, Cultural prejudices and ignorance prevent preparation for childbirth and obtaining assistance in many communities [13, 14]. This leads in development of unexpected complications due to delay in seeking care and spontaneous decisions [15–18]. To address these delays, the Johns Hopkins Program for International Education in Gynaecology and Obstetrics [JHPIEGO] developed the Birth Preparedness and Complication Readiness [BPCR] concept to ensure that pregnant mothers receive the care they require without undue delay [19]. These delays in decision making and receiving the right care can only be averted by better usage of the birth preparedness and complication readiness plan [17, 19, 20].

Birth preparedness and complication readiness [BPCR] demonstrates the expectant mothers on how to recognize labour symptoms, and warning indications of complications related to pregnancy. Additionally, it aids in lowering other obstacles to receiving care, such as transportation expenses, beliefs about the quality of care, and cultural differences [19, 21]. BPCR approach is an effective way to ensure the use of skilful maternal and neonatal services in a timely manner [22]. To have a BPCR plan in place helps to achieve the best potential outcome by being prepared for any unexpected issues that may arise during the birthing process [23]. BPCR strategies have significant impact on the utilization of skilled care and are successful in lowering maternity and fatality rates in resource-limited areas [23–25].

*BPCR facilitates the decision to seek care via two distinct approaches*. To begin, birth preparedness recommends planning on having a skilled professional present during labour. If this plan is put into action, the woman can get the necessary care before any potential issues arise during the birth, Consequently, the two delays are avoided. *The second step*, complication readiness enhances knowledge about danger signs in families and communities, increasing awareness of the problem and hastening the choice to seek treatment [26–28]. The Pradhan Mantri Surakshit Matritva Abhiyan in India, which was launched in 2016 has an objective mandating the BPCR plan for all the pregnant women with special emphasis on the women identified as having risk factor or with co-morbid condition [29].

Despite being a cost-effective strategy, BPCR is a neglected area of maternal healthcare in India. The existence of state-specific studies reporting the prevalence of BPCR, underscores the need for this study to get a national level BPCR estimates. Thus, our study aims to conduct a systematic review and meta-analysis on BPCR in India to understand the actual prevalence and practices of BPCR interventions in India. This systematic review and meta-analysis [SRMA] will provide a comprehensive overview of the current situation of BPCR in India. This analysis will bring to light the gaps in the existing knowledge base, identify potential areas of improvement, and inform evidence-based strategies to improve BPCR in India.

## Methodology

This review adhered to the guidelines laid down in the Cochrane Handbook of Systematic Reviews [30]. The protocol was registered at the International Prospective Register of Systematic Reviews [registration ID: CRD42023396109].

### Search strategy

Keywords were used to create a search strategy for addressing the research questions. Systematic search was performed by combining every feasible sequence of all the categories of keywords. The Medical Subject Headings [MeSH] terms and truncated keywords were mixed using the relevant Boolean logic operators i.e., AND, OR, and NOT. The authors [AKP and DG] pretested the search strategy in PubMed to ensure appropriateness in retrieving the relevant articles and subsequent modifications.

Based on the inclusion criteria, articles were retrieved from various search engines such as PubMed, Cochrane Library, and ProQuest. Additional articles were found by searching the reference lists of the identified studies in the Google Scholar. This review comprised all published papers up to January 31st, 2023. The following search phrases were used in the PICO format, as shown in Table 1. A detailed search terms were developed before the actual search for all the selected databases (Supplementary Table S1).

### Research question

What is the prevalence of BPCR among pregnant and recently delivered women in India?

**Table 1:** **PICO format**

Population	Pregnant women: “pregnan*“[Title/Abstract] OR “pregnant women“[Title/Abstract] OR “antenatal“[Title/Abstract] OR “anc“[Title/Abstract]
*Intervention*	-
*Comparison*	-
*Outcome*	Birth preparedness: “birth preparedness“[Title/Abstract] OR “preparedness“[Title/Abstract] OR “preparing for birth“[Title/Abstract] OR “emergency preparedness“[Title/Abstract] OR “birth plan“[Title/Abstract]
	Complication Readiness: “danger signs“[Title/Abstract] OR “readiness“[Title/Abstract] OR [[“recognisable“[All Fields] OR “recognise“[All Fields] OR “recognised“[All Fields] OR “recognises“[All Fields] OR “recognising“[All Fields] OR “recognize“[All Fields] OR “recognized“[All Fields] OR “recognizes“[All Fields] OR “recognizing“[All Fields]] AND “danger sign“[Title/Abstract]] OR “obstetric complication“[Title/Abstract] OR “pregnancy complication“[Title/Abstract] OR “obstetric danger sign“[Title/Abstract] OR “maternal complications“[Title/Abstract] OR “maternal health“[Title/Abstract] OR “newborn health“[Title/Abstract]

^a^ I and C are not mentioned as only descriptive studies were included

### Study selection and eligibility criteria

#### Inclusion criteria

This review covered cross-sectional studies on BPCR, conducted amongst pregnant & recently delivered women in India. All articles in English, regardless of time of data collection or publication year (taking 2009 as start year), were included.

#### Exclusion criteria

Studies that did not describe the study population or did not record the outcome variable as BPCR & studies reporting qualitative data were excluded.

### Selection process

Studies that satisfied the stated inclusion criteria were collected and independently examined by two reviewers [TS and BT]. For title and abstract screening, two reviewers separately assessed the eligibility of the studies received from the literature searches. A web-based automated screening application called “Rayyan.ai [31]” was used for duplicates removal, text mining and screening of records. Records were screened, labelled for inclusion, exclusion, or “maybe” relevance to the review’s subject, and the words for inclusion and exclusion were also highlighted [which significantly aided manual screening].

The generated reports from the two reviewers were retrieved and later full text were reviewed [TS and BT]. In cases of discrepancies, an agreement was reached by consensus with the advice of third-member arbitrators [AKP or DG]. Justification was given for excluding the studies with proper reason. The search process was presented in the form of a PRISMA flow chart.

### Data extraction and data collection

Using a predefined data extraction MS Excel spreadsheet prepared by the reviewer [AKP and DG], two reviewers [TS and BT] independently extracted the data. The Excel sheet included questions about the author’s name, publication year, study design, sample size, geographic location, participants, mean participant age, response rate, and the prevalence of BPCR. The tool also provides data on individual BPCR components such as proportion of women who saved money for childbirth and emergencies, prepared blood donors, identified skilled birth attendants, were aware of danger signs during pregnancy, labor, postpartum, and newborn, planned deliveries in medical facilities, arranged transportation, identified the place of birth, and were aware of government financial & transport assistance in JSY & JSSK.

### Main outcome

The main outcome of this review was to assess the prevalence of BPCR among pregnant and recently delivered women in India.

### Definition of BPCR

BPCR is a strategy to promote the timely use of skilled maternal and neonatal care, especially during childbirth, based on the theory that preparing for childbirth and being ready for complications reduces delays in obtaining this care. BPCR is measured by various key elements including arrangement for transportation, saving money for delivery, identifying skilled birth attendant, identifying place of delivery and identifying blood donor in the case of emergency, knowledge regarding danger signs during pregnancy, labour, postpartum and newborn. All studies that used the above definition of BPCR were included in this review [23, 32].

### Risk of Bias [ROB] Quality Assessment

Before identifying and finalizing the study for use, all studies were evaluated for methodological quality, risk of bias, and the validity of the study findings. The risk of bias of the included studies was independently appraised by two reviewers [TS and BT] using the Joanna Briggs Institute [JBI] [33] critical appraisal checklist, which is designed for research reporting prevalence data. The JBI methodology checklist consists of 9 questions and is categorized into “Yes”, “No”, “Unclear” and “Not applicable”.

Overall risk assessment was done by giving each study with a score between 0–9 and categorising them into any of the three categories of ROB: - [0–3 = HIGH, 4–6 = MEDIUM, and 7–9 = LOW]. Any disagreements over the assessment of the risk of bias and research quality between two reviewers was settled by involving the third reviewer/arbitrator [AKP or DG].

### Data synthesis, statistical analysis, and investigation of heterogeneity

The data was analysed using CMA [Comprehensive meta-analysis tool] and R studio software. The analysis was done by DG and AKP. We conducted a meta-analysis using the random effects models, taking into account the measured heterogeneity across the studies. The pooled estimates were reported by all the studies and the findings were presented using forest plots. Statistical heterogeneity was evaluated using a combination of visual inspection of the forest plot and assessment of the Cochran’s Q test, and the I^2^ statistic results, in reference to the Cochrane Handbook Criteria. A probability value of *p* < 0.05 was chosen to imply statistically significant heterogeneity. When the results fell below 25%, between 25% and 75%, and over 75%, heterogeneity was deemed low, moderate, and high respectively. When discrepancies between study outcomes go beyond those only due to chance, statistical heterogeneity was present. Funnel plot and Egger’s test were applied to explore the possibility of the publication bias in the studies. Sensitivity analyses were performed by removing the study(ies) with more than 10% weight and with extreme values (outliers) to determine the impact of individual studies on the pooled values and whether the aggregate estimates were dominated by a single study.

### Subgroup analysis

As per the data availability, we have done a subgroup analysis for BPCR by delivery status [recently delivered and pregnant women] and geographical region [East, West, North, South, and Central].

## Results

### Studies included in the review

**Fig. 1:**
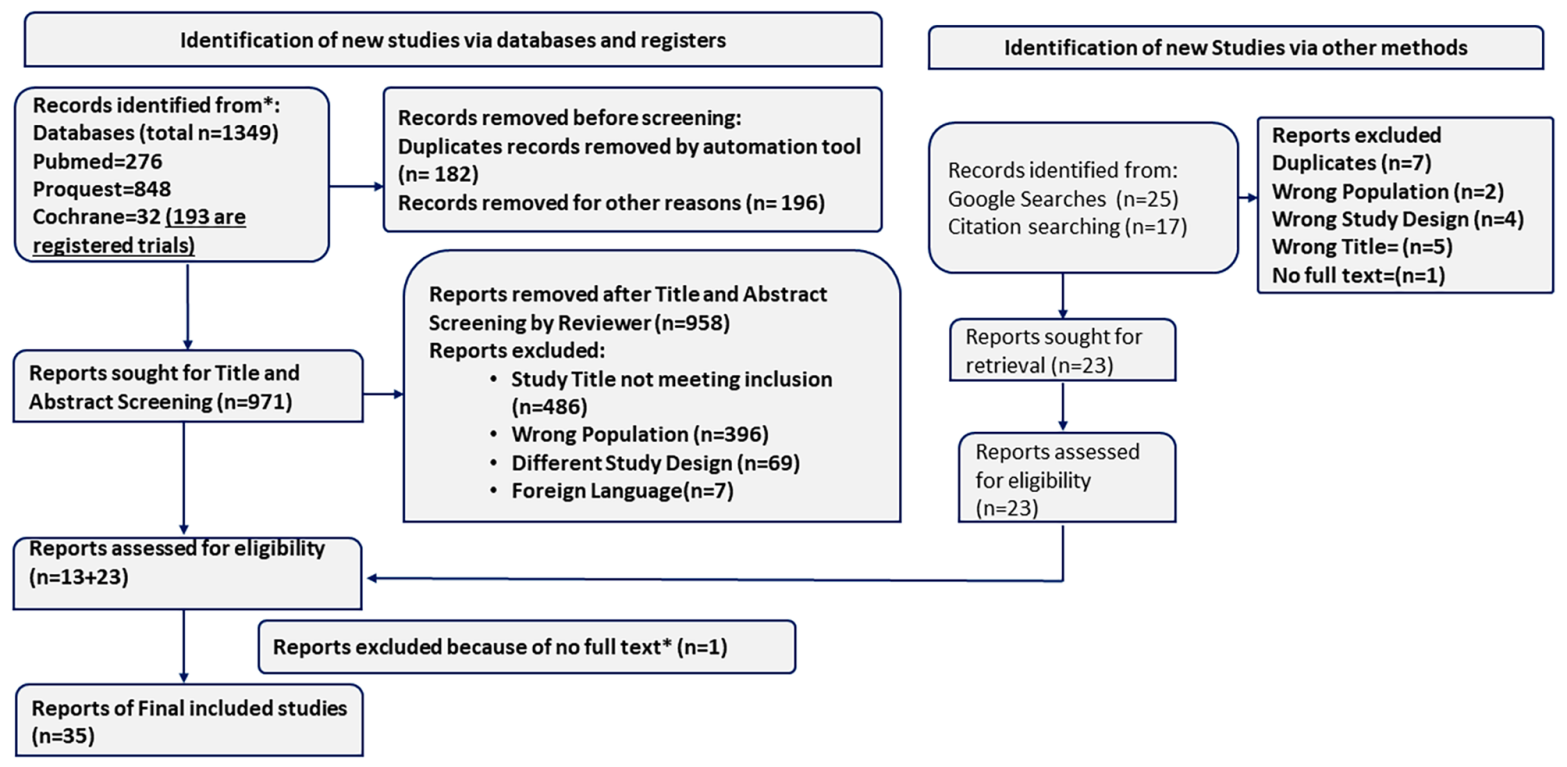
PRISMA diagram of the studies included in the meta-analysis

The initial search yielded 1391 articles, of which 1349 were from major databases like PubMed, Cochrane, and ProQuest, and 42 were from other sources like google searches and citation search. After removing duplicates and ineligible studies, 994 articles were screened for title and abstract screening, of which 36 articles were selected for evaluation on a full text screening. One study [34] was excluded due to unavailability of full text. Thus, in total, 35 studies were included in this review. PRISMA flow diagram showing studies’ selection process given in Fig. 1. To ensure scientific precision, MOOSE (Meta-analyses of Observational Studies in Epidemiology) checklist was also used (Supplementary Table S2).

### Study characteristics

All included articles were published in English and were conducted in various parts of India [Karnataka – 6; West Bengal − 5; Delhi, Gujarat, Madhya Pradesh – 3 in each state; Chhattisgarh, Maharashtra, Odisha, Tamil Nadu, Telangana, Uttar Pradesh – 2 in each state; Bihar, Haryana and Jammu & Kashmir – 1 in each state]. All studies followed cross-sectional study design. The studies were published between 2009 and 2022.

Out of these 35 studies, 16 studies [17, 21, 28, 35–47] collected data from pregnant women, 9 studies [24, 25, 27, 48–53]from recently delivered women, and 10 studies [22, 54–62] from both pregnant women and recently delivered women. All studies reported the information on birth preparedness and complication readiness except one study [60] which only took data on birth preparedness. Overall, 14,832 women either pregnant or recently delivered were the participants in the selected studies. Summary characteristics of studies included was prepared to provide greater insights (Supplementary file S3).

### Risk of bias [ROB] assessment

**Fig. 2:**
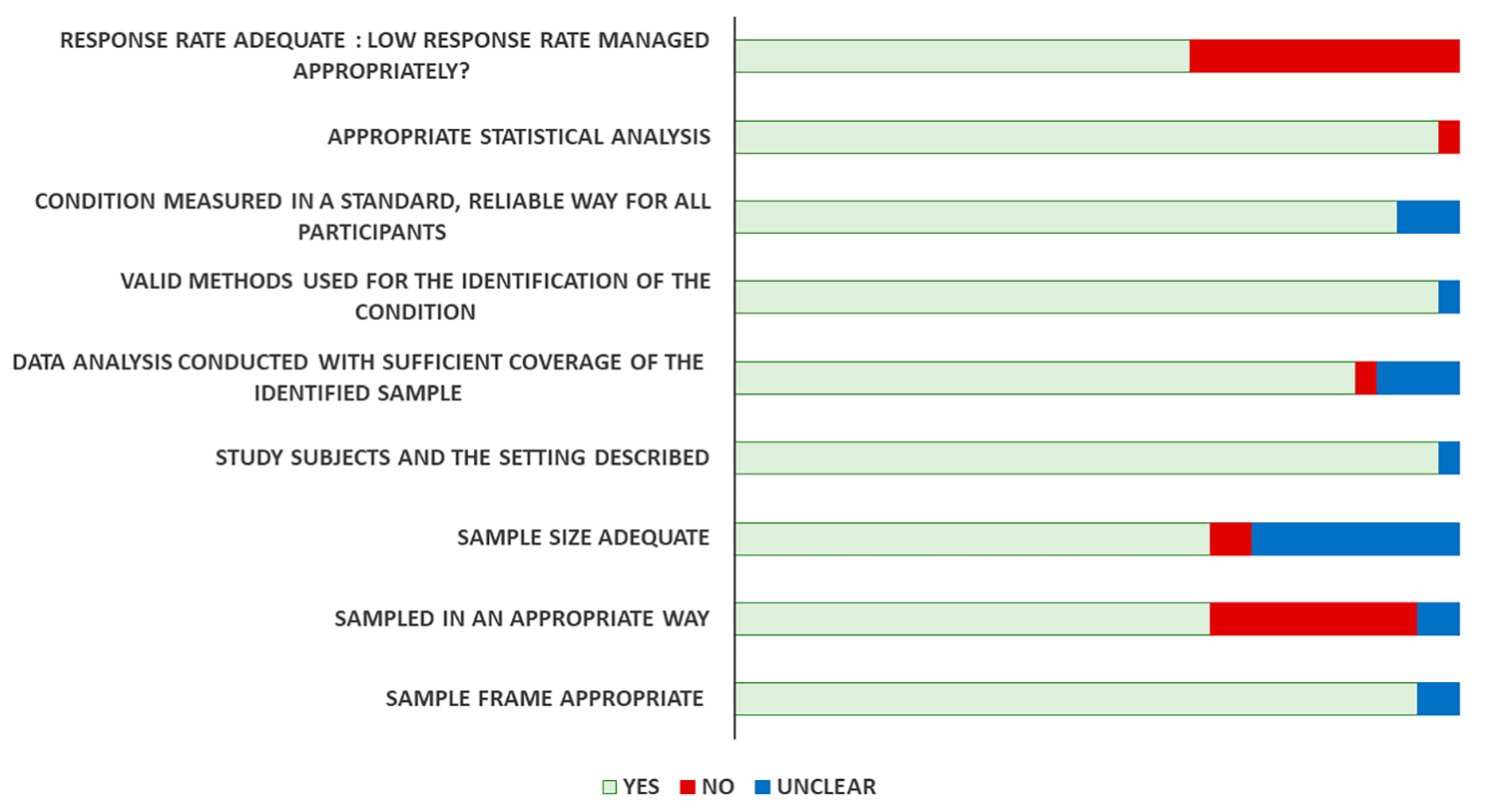
Summary of risk of bias for all included studies

The quality of different studies estimating the prevalence of BPCR included in this systematic review and meta-analysis was evaluated following the checklist proposed by the Joanna Briggs Institute (JBI) [33]. Studies with a score between “0–3” reporting “yes” indicated a “high” risk of bias, 4–6 indicated a “moderate” risk of bias, and a score of 7 or higher reporting “yes” belong to a "low" risk of bias. A summary of the risk of bias of the included articles is provided in Fig. 2. Of the total 35 included studies 28 [80%] fulfilled the criteria for low risk of bias, seven [20%] for moderate risk of bias and no study was found for high risk of bias. The results for risk of bias for each included study was also prepared to see individual study related risk of bias (Supplementary file as Table S4).

### Data synthesis

#### Overall BPCR

**Fig. 3:**
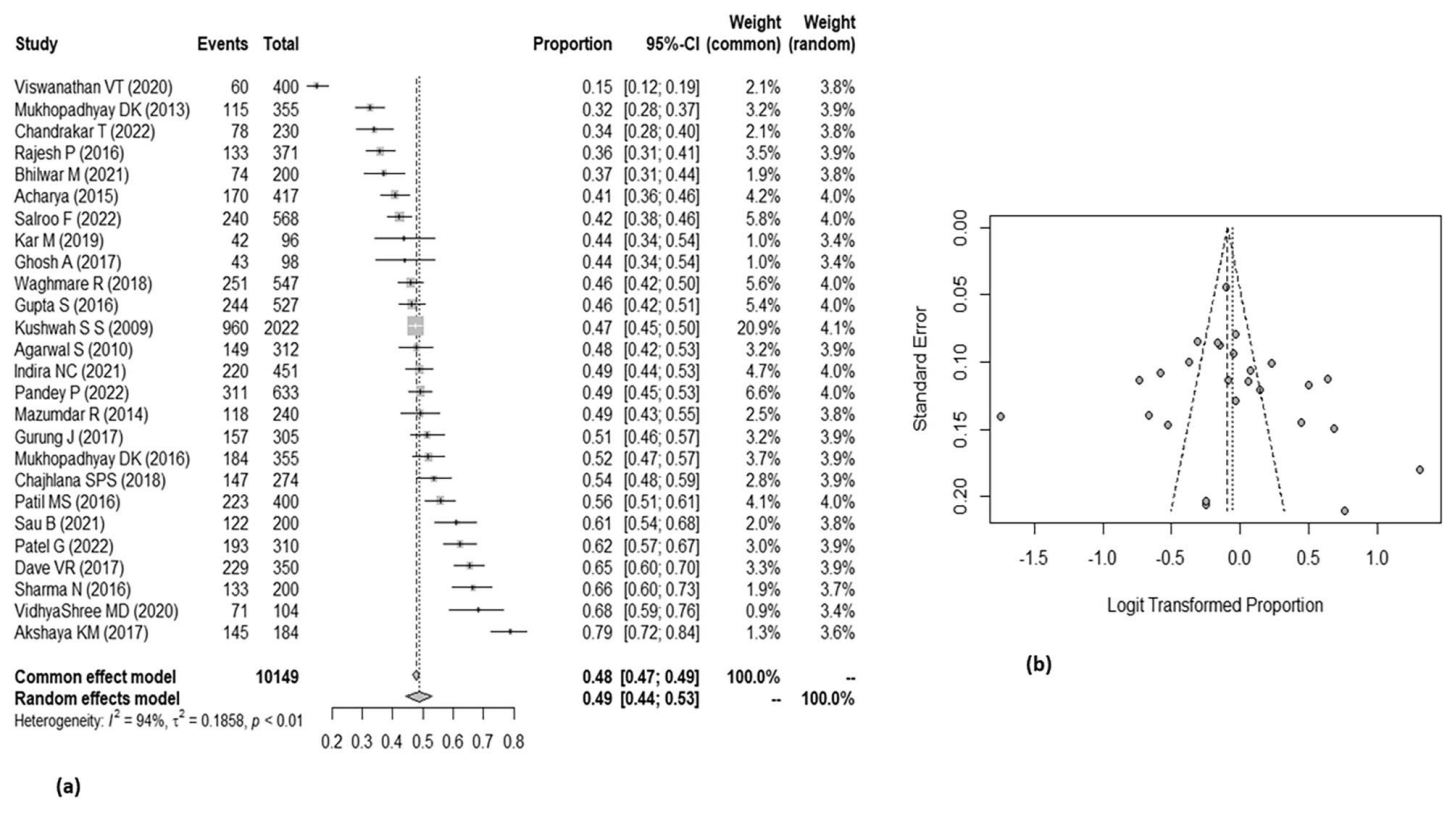
[**a**] Forest plot showing the prevalence of awareness of pregnant women or recently delivered women on BPCR. [**b**] Funnel plot showing distribution of studies on BPCR.

Out of 35 studies, overall knowledge on BPCR was given in 26 studies which were included in meta-analysis. Utilizing the random effect model, the pooled result showed that only half of the women [49%; 95% CI: 44%, 53%] were aware about birth preparedness and complication readiness. The minimum awareness was 15% [95% CI: 12%, 19%] reported by a study conducted by Viswanathan VT in Maharashtra, whereas the maximum was 79% [95% CI: 72%, 84%] in the study conducted by Akshaya KM in Karnataka [Fig. 3a].

The I^2^ test statistics result showed significant high heterogeneity [I^2^ = 94%, *p* = < 0.01]. Funnel plot shows symmetry in the studies [Fig. 3b], further confirmed by Egger’s test [*p*-value − 0.59], thus implying no publication bias. Performing the sensitivity analysis by removing the study with highest weight [56]and the extreme outlier studies [57, 58] did not make any significant difference in the pooled proportion [49%; 95% CI: 45%, 53%] and I^2^ [91%; *p*-value < 0.01].

### Sub-group analysis

Sub-group analysis was done based on the delivery status of women [pregnant women/recently delivered women], and region in which the study was conducted [East/West/North/South/Central].

#### Pregnant women vs recently delivered women

**Fig. 4:**
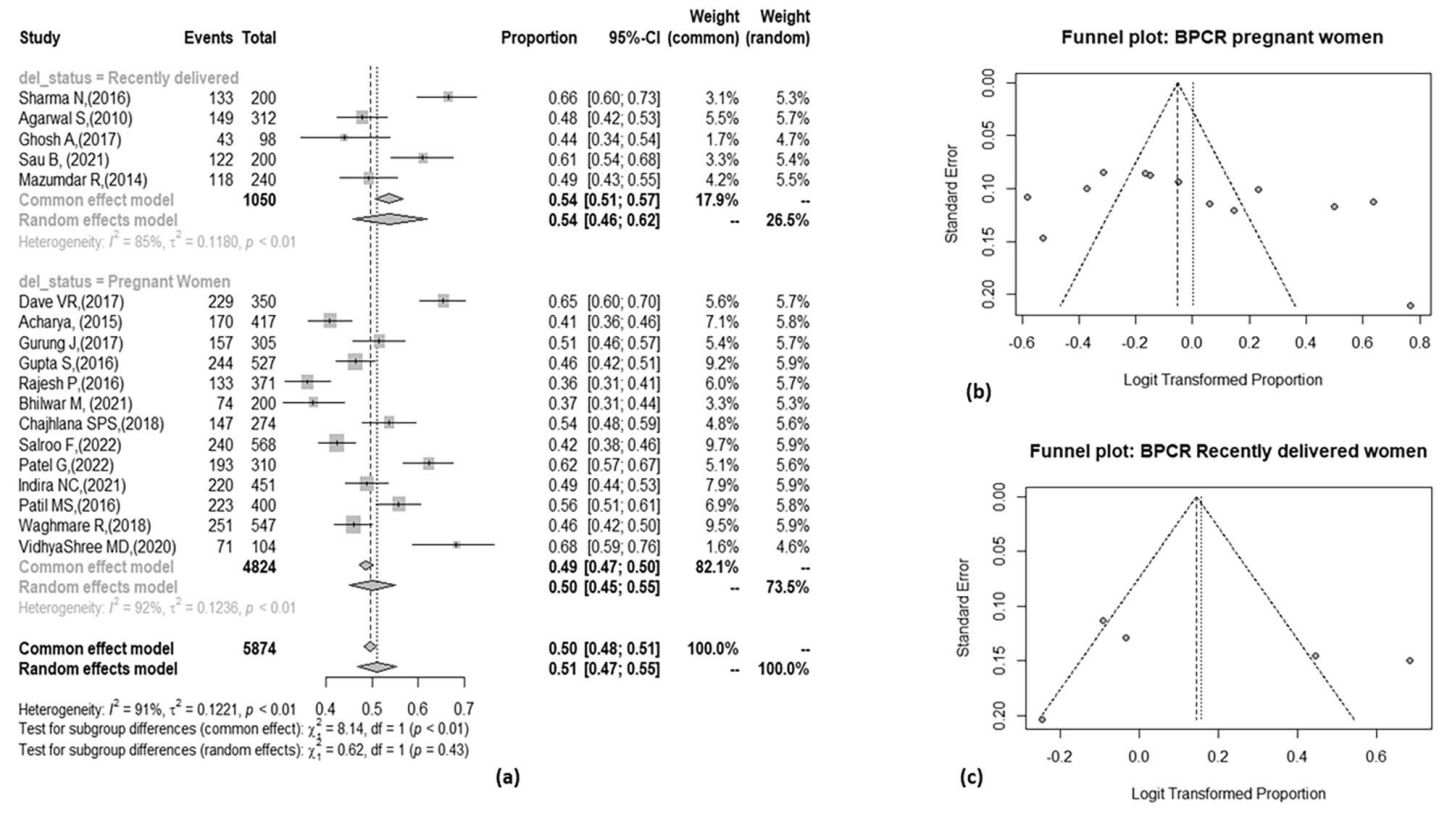
[**a**] Forest plot showing prevalence of BPCR among sub-groups of pregnant women and recently delivered women. [**b**] Funnel plot showing distribution of studies among pregnant women sub-group. [**c**] Funnel plot showing distribution of studies among recently delivered women sub-group

13 studies reported prevalence of BPCR among pregnant women distinctly and five among recently delivered women. Utilizing the random effect model, the pooled result showed no significant difference in the prevalence of BPCR among pregnant women [50%; 95% CI: 45%, 55%] and recently delivered women [54%; 95% CI: 46%, 62%]. The heterogeneity was high in both the groups with I^2^ being 85% [*p* < 0.01] among recently delivered women and 92% [*p* < 0.01] among pregnant women sub-group. Funnel plot for both the groups showed symmetrical distribution of studies. Egger’s test for pregnant women (more than 10 studies) confirmed no publication bias [*p*-value – 0.19] [Figure 4].

#### Study region

**Fig. 5:**
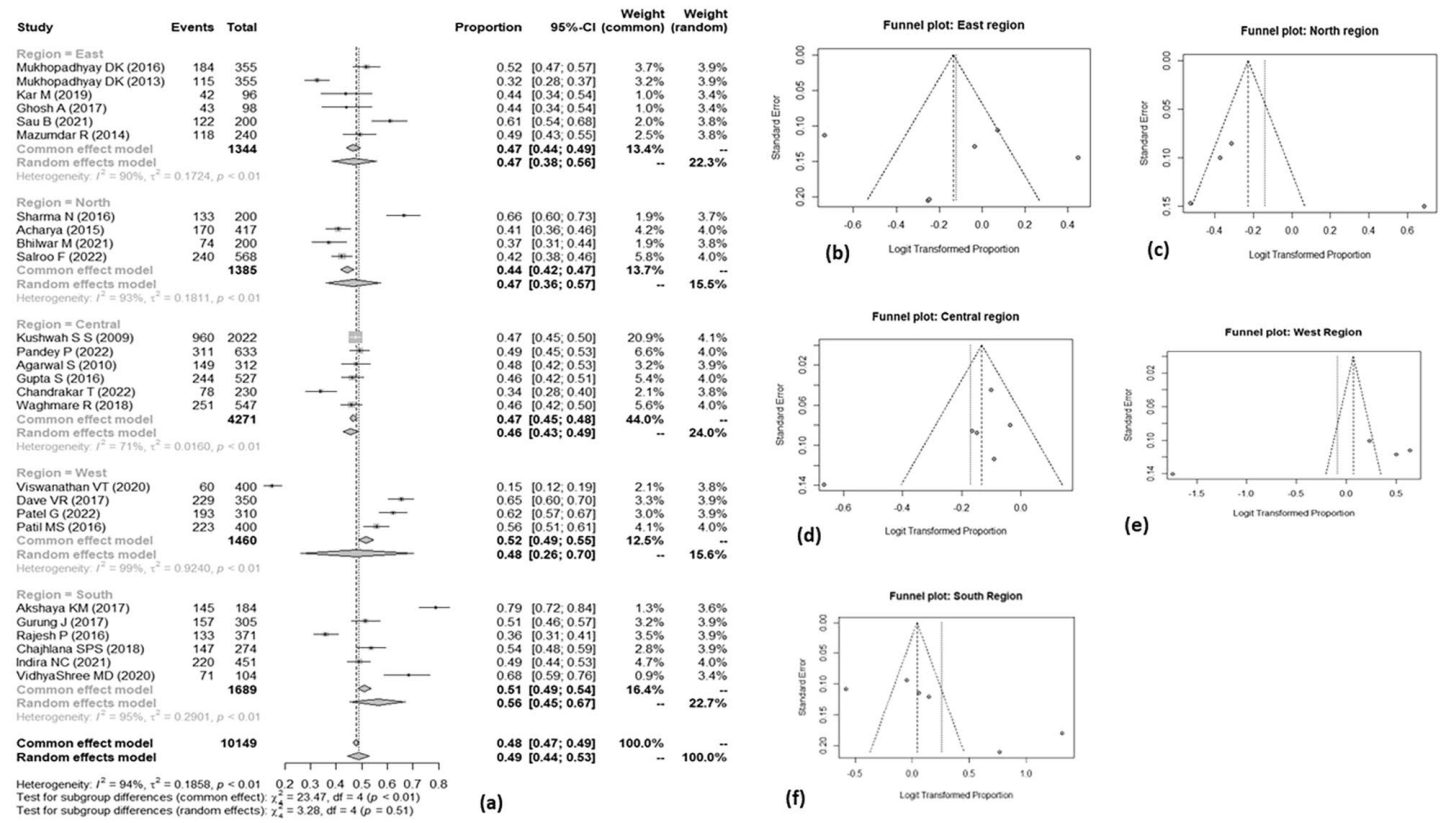
[**a**] Forest plot showing prevalence of BPCR among sub-group by different regions of India. [**b**], [**c**], [**d**], [**e**] and [**f**] Funnel plot showing distribution of studies among East, North, Central, West, South regions respectively

Out of total 26 studies, six studies each were conducted in east, central and south region of the country; and four each in west and north region. Utilizing the random effect model, the pooled result showed no major difference in the prevalence of BPCR among all the regions. South region showed relatively high [56%; 95% CI: 45%, 67%] prevalence as compared to other regions. The heterogeneity was significantly high in all the groups. The least heterogeneity was observed in central region as compared to other regions. Funnel plot for all the groups showed asymmetrical distribution of studies, thus indicating presence of publication bias [Figure 5].

### Components of birth preparedness and complication readiness

All the 35 studies reported knowledge on one or more components of birth preparedness, whilst knowledge on both complication readiness or danger signs was reported in 34 studies. Under knowledge of birth preparedness, maximum studies have reported the component of identifying transport in advance [*n* = 34], followed by saving money [*n* = 33], presence of skilled birth attendant [*n* = 22], and identification of blood donor in advance [*n* = 19]. Under knowledge of danger sign, maximum studies have reported the component of knowledge on only one danger sign during pregnancy [*n* = 13], followed by knowledge on only one danger sign of newborn [*n* = 8].

The average of the pooled result of all the components of birth preparedness and complication readiness depicts that knowledge on birth preparedness [61.63%] is much more than danger sign [22.05%]. Looking deeper into the components of birth preparedness [Table 1], the pooled prevalence of first antenatal visit with a skilled person during first trimester was highest [86.7%; 95% CI: 77%, 93%]. This is followed by knowledge on skilled birth attendant during delivery [83.3%; 95% CI: 73%, 90%]. Only about half of the women reported knowledge on place of delivery, transport through JSSK, financial assistance through JSY, identifying transport, save money, and four or more ANCs. Only 63% of women reported knowledge about early registration.

Among the components of danger sign, (Table 2), the pooled prevalence of knowledge on only one danger sign during pregnancy was highest [38.7%; 95% CI: 24%, 56%], followed by knowledge regarding danger signs during pregnancy [34.9%; 95% CI: 22%, 50%]. Only about one-third of women showed knowledge on only one danger sign of newborn, labour and postpartum and three danger signs of pregnancy. Less than 20% women reported knowledge on danger signs during labour, during postpartum, in neonates: three or more danger signs in neonates, during labour and postpartum period. Significant heterogeneity was high [> 90%] among all the components [*p* < 0.01].

**Table 2:** **Pooled result of knowledge on various components of BPCR**

Components		Events	Sample size	Prevalence	I^2^	TauSq	*P* value	Number of studies	LL^a^	UL^b^
Knowledge regarding Danger signs [DS]	Knowledge regarding danger signs during pregnancy	754	2,325	34.9%	97.4	0.95	0.00	10	0.22	0.50
	Knowledge regarding danger signs during labor	237	1,553	11.6%	98.0	1.69	0.00	5	0.04	0.29
	Knowledge regarding danger signs during postpartum	267	1,687	12.2%	91.1	0.32	0.00	6	0.08	0.19
	Knowledge regarding danger signs in neonate	195	1,189	18.8%	95.3	0.57	0.00	4	0.10	0.33
	3/more DS of newborn care	430	1,512	12.3%	97.6	0.88	0.00	4	0.05	0.27
	3/more DS of Postpartum	169	1,512	9.3%	94.2	0.66	0.00	4	0.04	0.20
	3/more DS of labor-childbirth	322	1,512	15.7%	92.7	0.30	0.00	4	0.09	0.26
	3/more DS of pregnancy	423	1,512	22.0%	93.6	0.26	0.00	4	0.14	0.33
	1 DS of newborn	837	2,770	28.8%	98.5	1.31	0.00	8	0.15	0.47
	1 DS of postpartum	567	1,863	30.4%	97.6	1.41	0.00	6	0.14	0.54
	1 DS of labor	649	2,367	30.0%	97.7	1.04	0.00	6	0.16	0.50
	1 DS of Pregnancy	1,559	4,339	38.7%	98.7	1.59	0.00	13	0.24	0.56
Knowledge regarding Birth preparedness [BP]	Place of delivery	2,192	5,964	59.4%	99.1	2.50	0.00	14	0.39	0.77
	Knowledge on Transportation	1,895	5,286	50.6%	99.0	1.32	0.00	11	0.34	0.67
	Financial assistance JSY	3,276	5,566	55.5%	98.7	1.06	0.00	13	0.41	0.69
	Skilled birth attendant	6,183	8,345	83.3%	98.6	1.86	0.00	22	0.73	0.90
	Identify blood donor	1,008	7,842	10.1%	98.0	1.45	0.00	19	0.06	0.16
	Identify transport	6,396	14,232	46.0%	98.8	1.28	0.00	34	0.37	0.56
	Saved Money	5,839	13,774	46.0%	98.8	1.28	0.00	33	0.40	0.56
	Four or more ANCs	2,205	4,910	51.5%	98.8	1.27	0.00	13	0.36	0.66
	1st antenatal visit with a skilled person during 1st trimester	1,958	2,283	86.7%	96.1	0.71	0.00	7	0.77	0.93
	Registration within12weeks	2,028	3,511	63.6%	99.0	1.37	0.00	8	0.44	0.80

a Lower limit

b Upper limit

## Discussion

The overall BPCR score of 49% in our study indicates that there is still a gap in the utilization of BPCR services among pregnant women in India. This score is slightly higher than the 44.9% reported by Girma et al. [2013] [63] in a similar study in Ethiopia. The variation in the BPCR scores may be attributed to the differences in the socio-demographic characteristics, health system factors, and cultural practices of the study populations. Compared to other studies in Nigeria, Ethiopia, and India by Akinwaare et al. [2015] [64], Gedefa et al. [2017] [65], and Nimavat et al. [2018] [66] respectively, our study had a moderate BPCR score of 49%, which ranged from 30.6% to 58.7% in these studies. Therefore, there is a need to improve the awareness and utilization of BPCR services among pregnant women, as well as to address the barriers and facilitators that influence their decision-making and behavior regarding BPCR.

The prevalence of women who saved money for their delivery was found to be 46% in our study, which is an important indicator of BPCR. This prevalence is higher than the 38.7% reported by Berhe et al. [2016] [32] in a study in Ethiopia, but lower than 63.4% reported by Akinwaare et al. [2015] [64] in Nigeria, 59.6% reported by Nimavat et al. [2018] [66] in India, and 83.3% reported by Moran et al. [2018] [67] in Burkina Faso. The differences in the prevalence of saving money for delivery may reflect the variations in the economic status, access to financial services, and social norms of the study populations. Saving money for delivery can help women to overcome the financial barriers to access skilled care during pregnancy and childbirth, and to cope with any unforeseen complications that may arise. Therefore, there is a need to promote and facilitate saving money for delivery among pregnant women, as well as to provide them with adequate information and counselling on the benefits and options of saving money for delivery.

The prevalence of women who have knowledge of key danger signs of pregnancy was 34.9% in our study, which is a low level of awareness that can compromise the timely recognition and management of pregnancy complications. This prevalence is higher than 26.3% reported by Berhe et al. [2016] [32] in a study in Ethiopia, but lower than 52% reported by Akinwaare et al [64]. [2015] in a study in Nigeria, 83.3% reported by Moran et al. [2018] [67] in a study in burkina faso, and 42% reported by Mukhopadhayay et al. [2016] [54] in a study in India. The differences in the prevalence of knowledge of key danger signs of pregnancy may be related to the variations in the educational level, exposure to mass media, and quality of antenatal care services of the study populations. Knowledge of key danger signs of pregnancy is essential for pregnant women to seek prompt and appropriate care when they experience any signs of potential complications, and to prevent maternal and neonatal morbidity and mortality. Therefore, there is a need to improve the knowledge on key danger signs of pregnancy among pregnant women, as well as to provide them with effective health education and counseling.

The prevalence of women who have arranged transport for their delivery was 46% in our study, which is a moderate level of preparedness that can facilitate the access to skilled care during childbirth. This prevalence is similar to 46.1% reported by Moran et al. [2018] [67] in a study in Burkina Faso, but varies from the other studies in Ethiopia, Nigeria, and India by Berhe et al. [2016], Akinwaare et al. [2015], Nimavat et al. [2018], and Mukhopadhyay et al. [2016] [32, 54, 64, 66], which reported prevalences ranging from 20.59 to 58.6%. The differences in the prevalence of arranging transport for delivery may depend on the availability, affordability, and acceptability of transport services in the study settings. Arranging transport for delivery can help women to overcome the geographical and financial barriers to reach a health facility in time, and to avoid delays and complications during delivery. Therefore, there is a need to encourage and support pregnant women to arrange transport for their delivery, as well as to improve the transport system and infrastructure in our area.

The prevalence of women who have identified a place of birth for their delivery was 59.4% in our study, which is a relatively high level of preparedness that can influence the choice and utilization of skilled care during childbirth. This prevalence is slightly higher than 54.85% reported by Berhe et al. [2016] [32] in a study in Ethiopia, which had a similar study design and population. The similarity in the prevalence of identifying a place of birth for delivery may reflect the common socio-cultural and health system factors that affect the decision-making and behavior of pregnant women in both settings. Identifying a place of birth for delivery can help women to plan ahead and to select a health facility that meets their needs and preferences, and to avoid uncertainty and confusion during delivery.

The prevalence of women who have arranged a blood donor for their delivery was 10.1% in our study, which is a very low level of preparedness that can increase the risk of maternal and neonatal mortality due to hemorrhage. This prevalence is similar to 8.18% reported by Berhe et al. [2016] [32] in a study in Ethiopia, and 9.9% reported by Mukhopadhyay et al. [2016] [54] in a study in India, but higher than 2.7% reported by Nimavat et al. [2018][63] in another study in India. The similarity in the prevalence of arranging a blood donor for delivery may indicate the common challenges and barriers that pregnant women face in accessing and obtaining blood transfusion services in these settings. Arranging a blood donor for delivery can help women to cope with any potential complications that may require blood transfusion, and to save lives during delivery. Therefore, there is a need to improve the prevalence of arranging a blood donor for delivery among pregnant women in our setting, as well as to improve the availability, accessibility, and quality of blood transfusion services in our area. Accredited Social Health Activist (ASHAs) in India plays a crucial role in knowledge and preparedness of pregnant women. Their active role might help pregnant women in various preparedness activities even for timely decision making to visit hospital for checkups and deliveries. However, the effectiveness of ASHAs in increasing maternal and newborn health care utilization and improving outcomes is mixed and should be studied separately.

We had multiple state specific studies reporting the prevalence of BPCR however we didn’t have a national level estimate thus this meta-analysis is one of its own kind of studies which has evaluated the pooled prevalence of birth preparedness and complication readiness in the country. We have strictly followed the guidelines laid down in the Cochrane book of systematic review. Despite several strengths, we do have limitations as we have restrained our search to articles published in English language and the last data searched was in January 2023. Due to variation in the selected study variables, we could not study the cultural, caste, and class differences on the prevalence of BPCR in India. Also studying effectiveness of ASHAs in strengthening the BPCR program was not carried out. Also, we did not address the variables influencing poor BPCR utilization, thus recommending researchers to undertake additional research in this area. To get an indepth understanding about the factors leading to inadequate knowledge on BPCR, we recommend undertaking a deep dive using the qualitative approach involving stakeholders from different levels and regions of country.

## Conclusion

Our study highlights the low prevalence of BPCR in India and the factors associated with it. Our findings underscore the need for targeted interventions to improve BPCR. Our study noted that there is a need to improve the awareness and utilization of BPCR services among pregnant women in India, as well as to address the factors that influence their decision-making and behavior regarding BPCR. Thus, consideration should be given to fortifying existing resources, such as frontline workers and primary healthcare, as a strategic approach to augmenting the effectiveness of awareness initiatives.

### Electronic supplementary material

Below is the link to the electronic supplementary material.

Supplementary Material 1

Supplementary Material 2

Supplementary Material 3

Supplementary Material 4

## Data Availability

All the data and literatures available in public were used for the systematic review and meta-analysis. The dataset can be obtained from one of the corresponding authors of the study stating the reason via mail on anuj.dr02@gmail.com, dikshagautam91@gmail.com. Request for full data could be made to the corresponding authors. Upon request we will provide access to full data.

## Abbreviations

BPCR: Birth Preparedness and Complication Readiness
GoI: Government of India JBI- Joanna Briggs Institute
JHPIEGO: Johns Hopkins Program for International Education in Gynaecology and Obstetrics
JSY: Janani Suraksha Yojana
JSSK: Janani Shishu Suraksha Karyakaram
LaQshya: Labour room Quality Improvement Initiative
MMR: Maternal Mortality Ratio
MMR: Maternal Mortality Rate
PMSMA: Pradhan Mantri Surakshit Matritva Abhiyan
PRISMA: Preferred Reporting Items for Systematic Review and Meta-Analysis
SRMA: Systematic review and meta-analysis WHO-World Health Organization
