# Supplementary material for: Examining birth preparedness and complication readiness: a systematic review and meta-analysis of pregnant and recently delivered women in India
Source: BMC Womens Health. 2024 Feb 14;24:119. doi: 10.1186/s12905-024-02932-4 (PMC10865639; doi:10.1186/s12905-024-02932-4)
Supplement: Supplementary file 1 — Supplementary Material 1 [file 12905_2024_2932_MOESM1_ESM.docx]

**S1 Table-A detailed search terms as per searched electronic databases**

| **Title** | | | | |
| --- | --- | --- | --- | --- |
| **Database** | **No** | **Search Query** | **Results** |  |
| **PUBMED (Data as on 06-02-2023)** | | | | |
|  | #1 | "pregnan*"[Title/Abstract] OR "pregnant women"[Title/Abstract] OR "antenatal"[Title/Abstract] OR "anc"[Title/Abstract] | **620,091** |  |
|  | #2 | "birth preparedness"[Title/Abstract] OR "preparedness"[Title/Abstract] OR "preparing for birth"[Title/Abstract] OR "emergency preparedness"[Title/Abstract] OR "birth plan"[Title/Abstract] | **20,978** |  |
|  | #3 | "danger signs"[Title/Abstract] OR "readiness"[Title/Abstract] OR (("recognisable"[All Fields] OR "recognise"[All Fields] OR "recognised"[All Fields] OR "recognises"[All Fields] OR "recognising"[All Fields] OR "recognize"[All Fields] OR "recognized"[All Fields] OR "recognizes"[All Fields] OR "recognizing"[All Fields]) AND "danger sign"[Title/Abstract]) OR "obstetric complication"[Title/Abstract] OR "pregnancy complication"[Title/Abstract] OR "obstetric danger sign"[Title/Abstract] OR "maternal complications"[Title/Abstract] OR "maternal health"[Title/Abstract] OR "newborn health"[Title/Abstract] | **39,433** |  |
|  | #4 | #1 AND #2 AND #3 | **276** |  |

| **Title** | | | | |
| --- | --- | --- | --- | --- |
| **Database** | **No** | **Search Query** | **Results** |  |
| **PROQUEST (Data as on 06-02-2023** | | | | |
|  | #1 | [noft(pregnan*) OR noft(pregnant women) OR noft(antenatal) OR noft(anc)](https://www.proquest.com/myresearch/savedsearches.checkdbssearchlink:rerunsearch/2311205/SavedSearches?t:ac=SavedSearches) | [**124,342**](https://www.proquest.com/recentsearches.recentsearchtabview.recentsearchesgridview.scrolledrecentsearchlist.checkdbssearchlink_0:rerunsearch/57058603E21E40ACPQ/None?t:ac=RecentSearches) |  |
|  | #2 | [noft(birth preparedness) OR noft(preparedness) OR noft(preparing for birth) OR noft(emergency preparedness) OR noft(birth plan)](https://www.proquest.com/myresearch/savedsearches.checkdbssearchlink:rerunsearch/2311204/SavedSearches?t:ac=SavedSearches) | [**29,912**](https://www.proquest.com/recentsearches.recentsearchtabview.recentsearchesgridview.scrolledrecentsearchlist.checkdbssearchlink_0:rerunsearch/5B2E6BA387A046C6PQ/None?t:ac=RecentSearches) |  |
|  | #3 | [noft(complication readiness) OR noft(readiness) OR noft(recognising danger signs) OR noft(danger sign) OR noft(obstetric complication) OR noft(pregnancy complication) OR noft(obstetric danger sign) OR noft(maternal complications) OR noft(maternal health) OR noft(newborn health)](https://www.proquest.com/myresearch/savedsearches.checkdbssearchlink:rerunsearch/2311203/SavedSearches?t:ac=SavedSearches) | [**95,890**](https://www.proquest.com/recentsearches.recentsearchtabview.recentsearchesgridview.scrolledrecentsearchlist.checkdbssearchlink_0:rerunsearch/DCBF1DEEA964AE3PQ/None?t:ac=RecentSearches) |  |
|  | #4 | #1 AND #2 AND #3  [(noft(pregnan*) OR noft(pregnant women) OR noft(antenatal) OR noft(anc)) AND (noft(birth preparedness) OR noft(preparedness) OR noft(preparing for birth) OR noft(emergency preparedness) OR noft(birth plan)) AND (noft(complication readiness) OR noft(readiness) OR noft(recognising danger signs) OR noft(danger sign) OR noft(obstetric complication) OR noft(pregnancy complication) OR noft(obstetric danger sign) OR noft(maternal complications) OR noft(maternal health) OR noft(newborn health))](https://www.proquest.com/myresearch/savedsearches.checkdbssearchlink:rerunsearch/2311207/SavedSearches?t:ac=SavedSearches) | [**848**](https://www.proquest.com/recentsearches.recentsearchtabview.recentsearchesgridview.scrolledrecentsearchlist.checkdbssearchlink_0:rerunsearch/BB9F3FE54E6142D2PQ/None?t:ac=RecentSearches) |  |
|  |  |  |  |  |

| **Title** | | | | |
| --- | --- | --- | --- | --- |
| **Database** | **No** | **Search Query** | **Results** |  |
| **Cochrane (Data as on 06-02-2023** | | | | |
|  | #1 | (pregnan*):ti,ab OR (pregnant women):ti,ab OR (antenatal):ti,ab OR (anc):ti,ab | **68406** |  |
|  | #2 | (birth preparedness):ti,ab OR (preparedness):ti,ab OR (preparing for birth):ti,ab OR (emergency preparedness):ti,ab OR (birth plan):ti,ab | **1639** |  |
|  | #3 | (complication readiness):ti,ab OR (readiness):ti,ab OR (recognising danger signs):ti,ab OR (danger sign):ti,ab OR (obstetric complication):ti,ab OR (pregnancy complication):ti,ab OR (obstetric danger sign):ti,ab OR (maternal complications):ti,ab OR (maternal health):ti,ab OR (newborn health):ti,ab | **14641** |  |
|  | #4 | #1 AND #2 AND #3 | **225** (193 -TRIALS, COCHRANE REVIEWS -**32**) |  |
