# Supplementary material for: Examining birth preparedness and complication readiness: a systematic review and meta-analysis of pregnant and recently delivered women in India
Source: BMC Womens Health. 2024 Feb 14;24:119. doi: 10.1186/s12905-024-02932-4 (PMC10865639; doi:10.1186/s12905-024-02932-4)
Supplement: Supplementary file 3 — Supplementary Material 3 [file 12905_2024_2932_MOESM3_ESM.docx]

**S3 Table. Summary characteristics of studies included for Meta-analysis.**

| **Author** | **Study setting** | **Sample Size** | **Inclusion** | **Outcome** | | **BPCR INDEX** |
| --- | --- | --- | --- | --- | --- | --- |
|  |  |  |  | **Birth preparedness** | **Complication readiness** |  |
| Mukhopadhyay DK, 2016 | West Bengal | n= 355 (PW=120, RDW= 235) | PW between 2nd and 3rd trimester; RDW within last 12 months, Permanent resident of the area | Registration within 12 weeks =83 (69.2), Saved money=52 (43.3), Identify transport=51 (42.5), Identified blood donor=11 (9.2), Awareness of government  financial assistance scheme=92 (76.7), Awareness of government  transport scheme=76 (63.3), BPCR index=45.2 | At least one key danger sign of pregnancy=43 (35.8), At least one key danger sign  of labor=32 (26.7) , At least one key danger sign  of postpartum=29 (24.2),At least one key danger sign  of newborn=46 (38.3),At least one key component  of essential newborn care=82 (68.3), | N=184, 52.1% |
| Kamineni V, 2017 | Hyderabad, | n=600(PW) | PW attending OPD at Hospital from oct 2012 to sept 2014. | registered in an ANC before 12 weeks of gestational age=84% (n=504), 77% (n = 462) were supervised by a qualified doctor before the visit to our hospital,did not identify a place of delivery (n=59),not started saving money(n=102),mothers  were not aware of purchasing materials needed for delivery(n=99),birth prepared (n=429) 71.5%, | no arrangements in the event of an emergency(n=164) 27%, n=376 (63%) were not aware ,no knowledge of the danger signs in pregnancy= 29%,signs of  severe conditions in labor=42%, serious health problems that  occur to newborns in the first 7 days of life=48%,20% (n = 120), 15.8% (n = 95), and 12% (n = 73) of  the respondents had knowledge of at least 3 dangers signs of  pregnancy, labor, and severe illness in newborn  of their blood group, 89% (n = 531) did not identify any  blood donor in case of any emergency. (n = 566) 94.3%, were not ready for any unexpected  emergencies during pregnancy,  the respondents had knowledge of at least 3 dangers signs of  pregnancy 20% (n = 120), labor 15.8% (n = 95), and severe illness in newborn 12% (n = 73) |  |
| Sharma N,(2016) | Haryana | n=200 (RDW) | RDW who have delivered a child in last 6 months. | Knowledge about financial assistance through JSY= 160 (80)  Knowledge of transportation provided by government through= JSSK 166 (83) ANC availed in first trimester from skilled provider= 155 (77.5)  Identified skilled birth attendant for delivery =189 (94.5)  Identified mode of transport =136 (68)  Saved money for expenses= 112 (56) | Knowledge of >8 danger signs= 19 (9.5) | N=133, 66.93 |
| Mukhopadhyay DK,(2013) | West Bengal | n= 355 (PW=117, RDW= 238) | PW between 2nd and 3rd trimester; recently delivered women within last 12 months, Permanent resident of the area | Registration within 12 weeks= 63 (53.8)  Saved money= 36 (30.8)  Identified transport =37 (31.7) Identified blood donor= 7 (6.0) | At least one key danger sign of pregnancy= 17 (14.5)  At least one key danger sign of labor =20 (17.1)  At least one key danger sign of postpartum =15 (12.8)  At least one key danger sign of newborn =34 (29.1)  At least one key component of essential newborn care =66 (56.4)  Awareness of Govt. financial assistance scheme =75 (64.1)  Awareness of Govt. transport scheme= 43 (36.8)  BPCR Index =32.1 | N=115, 34.5% |
| Kushwah S S,(2009) | Madhya Pradesh | n=2022( PW= 632, RDW=1390) | PW between 2nd and 3rd trimester,recently delivered women within last 12 months(1 year) | %of women who knew about transportation services provided by government under Janani Express Yojana=102(16.1), % of women availed ANC in 1st trimester by skilled provider=134(21.2),% of women who identified skilled birth attendant for delivery=481(76.1), % of women who identified mode of transportation=517(81.8), % of women who are saving money/ saved money to pay for expenses=259(41.0),% of women who knew about financial assistance provided in JSY=501(79.3), BP/CR Index= 47.0 | % of women who knew about >=8 danger signs of pregnancy= 87 (13.8), | n=960, 47.5% |
| Kusuma YS,(2018) | Delhi | n=458 (RDW) | migrant mothers with a child aged below one year of age . | Planned for hospital birth=194 (42.4) Planned for transportation to health facility=140 (30.6) Aware of government transport services=75 (16.4) Utilized government ambulance=8 (1.7) Saved money for childbirth =267 (58.3) | Knowledge of danger signs (Knew at least one)= 60 (13.1) |  |
| Viswanathan VT,(2020) | Maharastra | n=400 PW And RDW | PW women in 3 rd trimester and RDW upto 7 days | 6. Registration of pregnancy =378 (94.5) 7. Planning 4 or more ANC visits= 376 (94) 8. ANC visit in first trimester =386 (96.5) 9. TT first dose =389 (97.3) 10. 100 iron and folic acid consumed =384 (96) 11. Awareness regarding the possible need for blood transfusion during delivery=38 (9.5) 12. Saved money for childbirth= 180 (45)  13. Identified mode of transport =252 (63) 14. Ideal postnatal visits =316 (79) 15. Immunization= 400 (100) 16. Need for the birth companion =261 (65.3) 17. Knowledge regarding JSSK= 134 (33.5) 18. Knowledge regarding transport service under JSY= 145 (36.3) 19. Knowledge regarding Vandemataram scheme= 40 (10) 20. House visits from ASHA =340 (85) 21. Planning birth with a skilled provider =387 (96.8) | 1.Knowledge regarding danger signs during pregnancy=87 (21.8) 2. Knowledge regarding danger signs during labor =17 (4.3) 3. Knowledge regarding danger signs during postpartum=38 (9.5) 4. Knowledge regarding danger signs in neonate 37 (9.3) 5. Urgency in seeking hospital care for danger signs= 397 (99.3) | n=59.56 ,52% |
| Pandey P,(2022) | Uttar Pradesh | n=633, (PW=336 & RDW=297) | Pw in 2nd and 3rd trimester & RDW within last 6 months | 1st ANC check‑up done in 1st trimester =135 (40.2),BPCR index=∑11 indicator (%)/11 =49.2 (total) Knowledge about minimum 4 ANC check up= 85 (25.3)  Knowledge about government financial assistance =313 (93.2)  Knowledge about government ambulance service =308 (91.7)  Identified a skilled provider/health facility =320 (95.2)*  Saved money =184 (54.8)  Arranged a transport =62 (18.5) | Knowledge of ≥3 danger sign of pregnancy =124 (36.9)  Knowledge of ≥3 danger sign of labour= 110 (32.7)  Knowledge of ≥3 danger sign of postpartum period= 25 (7.4)  Knowledge of ≥3 danger sign of new born =154 (45.8) | n=311, 49.2% |
| Dave VR,(2017) | Gujarat | n=350, (PW) | antenatal women | Identified place for delivery=86.57% Identified Transportation Mode when in need=76.29% Identified Decision Taker in case of emergency=88.29% Identified Place for emergency referral=82.29% Identified doctor for new bom=16.29% Saved money for pregnancy related expenses=52.57% | Knowledge of Danger signs during Pregnancy =8.00% Knowledge of Symptoms of True labour=9.43% Danger signs of labour=41.43% Knowledge of Danger signs during post-partum period =20.57% | n=229 65.43% |
| Akshaya KM,2017 | Karnataka | n=184 (60 pregnant and 124 recently delivered) | PW (>28 weeks) and RDW (in the last 6 months) | women had 4 ANC visits during their pregnancy=78.3% (n = 144) aware of JSY=47.9% (n = 69) adequately informed about BPCR by a doctor/ANM/ASHA=(n = 114, 62%) Optimal BPCR practice was observed in (n=145, 79.3%) of the women. identified the place of delivery (n = 184, 100%),  saved money to pay for expenses (n = 96, 52.2%), identified the mode of transport to the place of childbirth (n = 132, 71.7%),  identified a birth companion (n = 167, 90.8%)  arranged a blood donor if the need arises (n = 29, 15.8%). women were accompanied by her husband/mother/mother in-law for ANC visits=158 (85.9%) | women were knowledgeable on key danger signs=(n=98, 53.8%). Aware of at least one danger sign (n=147 ,79.9)  Aware of at least six danger signs (n=99 ,53.8) Public health sector =97 (52.7) Private health sector =87 (47.3) | n=145, 79.3% |
| Acharya, (2015) | Delhi | n=417(PW) | antenatal women residing at the study  area | ANC registration and care in first trimester by skilled provider= 179 (42.9)  Identified skilled birth attendant for delivery= 338 (81.1)  Identified mode of transportation =184 (44.1)  Saved money to pay for expenses= 204 (48.9) | Overall, one-fifth (27.8%) women knew about anyone danger sign of pregnancy  The most common danger signs known were severe bleeding (20.1%)  followed by pain abdomen (8.6%)  swelling of face and hands (6.7%),  and reduced fetal movement (5.8%).  Only 28 (6.7%) knew about danger signs in labor  only 3 (0.7%) knew about danger sign during puerperium | n=170, 41% |
| Agarwal S,(2010) | Madhya Pradesh | n=312(RDW) | RDW having infants aged 2-4 months | Identified a trained birth attendant for delivery= 217 (69.6),Arranged for transport= 92 (29.5), Saved money =240 (76.9).Identified a health facility for emergency =199 (63.8) | Aware of at least one dange sign during pregnancyr= 83.9%,Aware of at least one danger sign during delivery=83.2 |  |
| Gurung J,2017 | Karnataka | n=305 | Pregnant women who were in the second and third trimesters | Identified a skilled birth attendant= 302 (99) Identified a facility for emergency= 129 (42.3) Identified blood donor= 3 (1) Arranged transport 305 (100) Saved money 80 (26.2) | Awareness of at least one danger-sign During pregnancy 156 (51.1) During delivery 85 (28) Newborn danger sign 97 (32) | n=157, 51.47% |
| Gupta S ,2016 | Uttar Pradesh | n=527 | pregnant women were serially included in the study who attended antenatal clinic of PHC. | Women who knew about financial assistance provided by government in Janani Suraksha Yojana (JSY)=47% Women who knew about transportation provided by government in JSY =27% Women who availed Antenatal Care (ANC) in 1st trimester by skilled provider=44% Women who identified skilled birth attendant for delivery=83% Women who identified mode transportation=41% Women who saved money to pay for expenses.=45% | Women who knew about > 8 danger sign of pregnancy=38% | n=244, 46.4% |
| Kar M ,2019 | Odisha | n=96 | pregnant women who completed 24 weeks of gestation and women who had delivered recently within last 12 months preceding date of survey regardless of newborn outcome. | Govt. financial scheme  (JSY)=68 (70.8) Location for emergency  care=45 (46.9) Delivered (plan to) with SBP=80 (83.3) Saved (plan to) money=44 (45.8) Identified (plan to) vehicle for emergency transportation=25 (26) Identified (plan) blood donor=14 (14.6) | Key one danger sign in pregnancy=13 (13.5) Key danger sign in Delivery=7 (7.3) Key danger sign in Postpartum=13 (13.5) Key danger sign in newborn=20 (20.8) | n=42 (44.6%) |
| Karir S ,2022 | Odhisha | n=111 | All those who had delivered during the reference period of the preceding year, i.e., from first day of January of 2019 till the last day of December of 2019 were included in the study | Identified mode of transport=38 (34.2) Identified a trained birth  attendant=65 (58.5) Saved money=45 (40.5) Identified a medical facility =77 (69.4 ) Identified a blood donor =10 (9.0) | Knowledge of at least 3 key danger signs of pregnancy=34 (30.6) Knowledge of at least 3 danger signs in labour=12 (10.8) Knowledge of at least 3 key post-partum danger signs =16 (14.4) Knowledge of at least 3 key danger signs in newborn=2 (1.8) |  |
| Rajesh P et al,2016 | Karnataka | n=371 | pregnant women who attended antenatal clinic between June-September 2015 in the district hospital Tumkur. Karnataka. Tumkur district hospital | Percentage of women who attended 1st antenatal visit with a skilled person during st trimester=97.3% Percentage of women who plan to give birth with a skilled provider=99.54% Percentage of women who plan to identify a mode of transport to the place of delivery=28% Percentage of women who plan to save money for child birth=11.42% Percentage of women who knew about the financial assistance under Janani Suraksha Yojana=60% Percentage of women who knew about the transport assistance under Janani Suraksha Yojana=36.39% | Percentage of women who knew at least 8 key danger signs during pregnancy, labour & during postpartum period | n= 133,35.85% |
| Rakesh J ,2017 | Karnataka | n=200 | women attending a rural maternity at Ramanagara taluk in southern Karnataka for delivery were interviewed | Identified place of delivery=144 (72) Identified mode of transport=68 (34) Saved money =200 (100) Identified a blood donor=0 (0) | Key danger signs during  pregnancy (%)=131(65.5) Key danger signs during labour (%)=41(20.5) Key danger signs after  childbirth (%)=42(21) Key danger signs in newborn (%)=88(44) |  |
| Bhilwar M, 2021 | Delhi | n=200 | Pregnant women irrespective of their gestational age and parity who gave written consent to participate | Arranged a mode of transport =57 (28.5) Arranged a blood donor =12 (6.0) Saved money for pregnancy and childbirth =106 (53.0) Identified an institute for delivery 122 (61.0) | Awareness about at least one key danger sign of pregnancy =26 (13) Awareness about at least one key danger sign of labor =28 (14) Awareness about at least one symptom indicating onset of labor=60 (30 ) Awareness about at least one government cash scheme =41 (20.5) | n=74.24,37.12 |
| Shastri VD ,2019 | Bihar | n=2366 | Both PW and RDW | Saved money= 184 , identified transport=45 , Identified Blood donor=14,Identified place for delivery=50 |  |  |
| Chajhlana SPS,2018 | Telangana | n=274 | pregnant women who attended antenatal clinics between May 2014 –Aug 2014 at rural health and training center | Study subjects who attended 1st antenatal visit with a skilled person during 1st trimester= 218 (79.6) Study subjects who plan to give birth with a skilled provider= 213 (77.7) Study subjects who planned place of delivery= 188 (68.6) Study subjects who plan to identify a mode of transport to the place of delivery= 201 (73.4) Study subjects who plan to save money for child birth= 212 (77.4) Study subjects who knew about the financial assistance under Janani Suraksha Yojana =208 (75.9) Study subjects who knew about the transport assistance under Janani Suraksha Yojana =187 (68.2) | Study subjects who knew at least eight key danger signs during pregnancy, labour & during postpartum period=0 (0) Study subjects who knew at least one key danger signs during pregnancy, labour & during postpartum period=223 (81.4) Study subjects who knew at least four key danger signs during pregnancy, labour & during postpartum period=148 (54.0) | n=147, 54% |
| Chandrakar T et.al,2022 | Chattisgarh | n=230 | Pregnant women in the 3rd trimester residing in that area for a minimum period of 6 months and Women delivered within 6 months’ duration from the date of survey | Percentage of women who attended 1st antenatal visit with a skilled person during 1st trimester= 124 (53.9) Percentage of study participants who went for attending or planned to attend at least 4 ANC visits=178 (77.4) Percentage of study participants who either gave birth or planned to give birth with a trained health service provider=87 (37.8) Percentage of study participants who saved or planned to save money for delivery=35 (15.2) Percentage of study participants who have earmarked or planned to earmark a transport modality to health facility=81 (35.2) | Percentage of study participants who could recollect and tell all three key danger signs of pregnancy period=0 Percentage of study participants who could recollect and tell all four key  danger signs of labour=0 Percentage of study participants who could recollect and tell all three key danger signs of post‑partum period=0 Percentage of study participants who could recollect and tell all four key  danger signs in newborn=0 | n=78 (34.1%) |
| Sulekha T et.al,2020 | Karnataka | n=100 | From all the ‘anganwadis’ (government health centres for women and children) in the two sub-centers, we obtained a list of 146 mothers who had delivered over the previous year. | Identify the health facility for delivery=49 (49) Identify emergency transport= 27 (27) Save money for the delivery =33 (33) Identify a potential blood donor =0 (0) | The overall knowledge of danger signs in pregnancy, labour, and postpartum was found to be 18%, 0%, and 4% respectively |  |
| Salroo F,(2022) | Jammu and Kashmir | n=568 | PW who are willing to participate & attending the OPD of O&G of GMC, Anantnag | Women who attended first ANC visit with a skilled provider during first trimester=555 (97.7) ,Women who arranged transport to the place of childbirth  =441 (77.6),Women who identified a skilled birth  attendant for this childbirth to give birth with =62 (10.9),Women who saved money for childbirth=431 (75.9),Women who identified a blood donor for this  childbirth =323 (56.9) | Women who had knowledge about three key danger signs during pregnancy  =161 (28.3),Women who had knowledge about four key  danger signs during labor and childbirth=120 (21.1),Women who had knowledge about three key danger signs during postpartum period=112 (19.7),Women who had knowledge about four key  danger signs in the newborn=138 (24.3) |  |
| Ghosh A,(2017) | West Bengal | n=98 | RDW given birth during June 2014-May 2016. | Arrangement a skilled birth attendant=98 (100),saved mooney for emergency= 54 (55.1),Arranged for transport= 33 (33.7), | Knowledge regarding danger signs during pregnancy=32, Knowledge regarding danger signs in neonate=44 |  |
| Sau B, (2021) | West Bengal | n=200 | RDW given birth within last 7days | Women who identified Skilled Birth Attendant (SBA) for  delivery =200 (100),Women who identified mode of transportation=77 (38.5),  Women who saved money to pay for expenses= 76 (38.0) | Women who knew more than eight Danger Signs of pregnancy =0 |  |
| Patel G,(2022) | Gujarat | n=310 | Pw attending antenatal clinic of UHTC OPD. | ANC Registration=300,4 or more ANC visits=212,Identified skilled birth attendant=271,Identified mode of transportation=238,Saved money=215,Identification of blood donor=32 | knew at least one key danger sign of pregnancy=98.06%,know at least one key danger sign of labour=99.35% |  |
| Mutreja S,(2015) | Chhattisgarh | n=146 | PW & RD women who had delivered a child 12 months prior to May – June 2013. | Knowledge of expected date of delivery, knowledge to save money for emergency need, identified  place of birth, arrangement for transport, identified skilled attendant, identified birth companion were= 31.9%, 31.9%, 34.7%, 26.4%, 13.9% and 77.8% among PW and 37.8%, 14.9%, 32.4%, 16.2%, 14.9% and 68.9% among RDW. | Knowledge of danger signs during pregnancy, childbirth and post partum period was = 30%, 9% and 4%,knowledge of at least three danger signs=21.2% |  |
| Indira NC,(2021) | Tamilnadu | n=451 | all PW who visited antenatal clinic  during May to August 2019 | Identified mode of transport= 320( 71), Saved money in case for emergency=370 (82) Identified blood donor= 120 (26.6), Identified skilled birth attendant= 17( 3.8), Identified place of delivery =274 (60.8) | Knowledge about danger signs in pregnancy=146(32.4) Knowledge about danger signs of postnatal period=106 (23.5) Knowledge about danger signs of newborn=81 (18) Knowledge about BPCR= 220 (48). | n=220 ,48.8% |
| Patil AA,(2022) | Karnataka | n=252 | PW present in the house at the time of visit and willing to share information. | Identify the mode of transport= 135 (54), Identify blood donor= 82 (33) ,Identify hospital for delivery= 211 (84),  Identify skilled provider=40 (16),Saved money= 111 (44),  Know the blood group= 190 (75) | Adequate & complete knowledge of danger signs (>6)= 78(31) Adequate & complete knowledge of danger signs (>3) =135(53) |  |
| Patel NA,(2017) | Gujarat | n=450 | RDW  who had delivered in last one year (1st September 2009 to 31st August 2010) | Identified place of delivery and attendant=344 (76.44) Saved money= 169 (37.56) Prepared vehicle =70 (15.56) | women had knowledge regarding all the danger signs=7 (2.5%),Knowledge of women regarding danger signs  of pregnancy (n=278) |  |
| Patil MS,(2016) | Maharastra | n=400 | PW who gave consent and attended ANC OPD of SRT from August- December 2015 | Identified skilled birth attendant for delivery= 393 (98.14), Registration of ANC and care in first trimester by skilled provider=319( 79.75), Identified mode of transportation= 289( 72.25), Knowledge about transportation provided through JSSK= 252 (63.00), Knowledge about financial assistance provided through JSY=185 (46.25), Saved money for expenses =126 (31.48) | women knew about any one danger signals of ANC=163 (40.75%) | n=223 ,55.83% |
| Waghmare R,(2018) | Madhya Pradesh | n=547 | PW attending the anganwadi seeking care at RHTC, manglia and those who gave consent | Registration within 12 weeks=53 Four or more ANCs= 46 Institutional deliveries= 102 Saved Money=129 Identify transport=125 Identified blood donor=79 Women who knew about financial assistance provided by government in Janani Suraksha Yojana (JSY)= 132 Women who knew about transportation provided by government in JSSK/jsy=97 | At least one key danger sign of pregnancy= 18 At least one key danger sign of labor=17 At least one key danger sign of postpartum= 12 At least one key danger sign of newborn= 11 At least one key component of essential newborn care=31 | n=251 ,46.02 |
| VidhyaShree MD,(2020) | Tamilnadu | n=104 | PW second and third trimester attending urban health centre field practice area under Pudupet | Saved money= 49 , identified transport=39 , Identified Blood donor=14 | Knowledge regarding danger signs during pregnancy= 94 |  |
| Mazumdar R,(2014) | West Bengal | n=240 | RDW who delivered in last 12 months. | Percentage of women received first  antenatal check-up within first trimester=83.8% ,Percentage of women received 4 ANCs= 87.9% ,Percentage of women delivered with SBA= 90% ,Percentage of women saved money for childbirth= 84.6% ,Percentage of women identified vehicle for emergency transportation=70.4% ,Percentage of women identified blood donor=12.9% | Percentage of women knew key danger signs of pregnancy=18.8% ,Percentage of women knew key danger  signs of labor/ childbirth=14.2% ,Percentage of women knew key danger  signs of post-partum period=17.5% ,Percentage of women knew key danger signs of newborn= 13.8% | n=118, (49.4) |
