# Supplementary material for: Examining birth preparedness and complication readiness: a systematic review and meta-analysis of pregnant and recently delivered women in India
Source: BMC Womens Health. 2024 Feb 14;24:119. doi: 10.1186/s12905-024-02932-4 (PMC10865639; doi:10.1186/s12905-024-02932-4)
Supplement: Supplementary file 4 — Supplementary Material 4 [file 12905_2024_2932_MOESM4_ESM.docx]

| **S4 Table**. Quality assessment of included studies using JBI quality assessment tool | | | | | | | | | | | | |
| --- | --- | --- | --- | --- | --- | --- | --- | --- | --- | --- | --- | --- |
| **Author, year** | **Study** | **Was the sample frame appropriate to address the target population** | **Were study participants sampled in an appropriate way?** | **Was the sample size adequate?** | **Were the study subjects and the setting described in detail?** | **Was the data analysis conducted with sufficient coverage of the identified sample?** | **Were valid methods used for the identification of the condition?** | **Was the condition measured in a standard, reliable way for all participants?** | **Was there appropriate statistical analysis?** | **Was the response rate adequate, and if not, was the low response rate managed appropriately?** | **Total Yes** | **overall risk 0-3=HIGH, 4-6=MEDIUM, 7-9=LOW** |
| Mukhopadhyay DK, 2016 | Birth preparedness and complication readiness among women of Bankura District, West Bengal | Yes | Yes | Yes | Yes | Yes | Yes | Yes | Yes | Yes | 9 | LOW |
| Kamineni V,2017 | Birth preparedness and complication readiness in pregnant women attending urban tertiary care hospital | Yes | No | No | Yes | No | Yes | Yes | No | No | 4 | MEDIUM |
| Mukhopadhyay DK,2013 | Status of birth preparedness and complication readiness in Uttar Dinajpur District, West Bengal | Yes | Yes | Yes | Yes | Yes | Yes | Yes | Yes | Yes | 9 | LOW |
| Kushwah S S,2009 | Status of birth preparedness & complication readiness in Rewa District of Madhya Pradesh | Yes | Yes | Yes | Yes | Yes | Yes | Yes | Yes | No | 8 | LOW |
| Pandey P ,2022 | Status of Birth Preparedness and Complication Readiness of Pregnant Women and Recently Delivered Women in Rural Varanasi: Assessment of Current Scenario | Yes | Unclear | Yes | Yes | Yes | Yes | Yes | Yes | Yes | 8 | LOW |
| Dave VR,2017 | Assessment of the birth preparedness and complication readiness among antenatal women at Ahmedabad city, India | Yes | Yes | Yes | Yes | Yes | Yes | Yes | Yes | Yes | 9 | LOW |
| Akshaya KM,2017 | Birth preparedness and complication readiness among the women beneficiaries of selected rural primary health centers of Dakshina Kannada district, Karnataka, India | Yes | Yes | Yes | Yes | Yes | Yes | Yes | Yes | Yes | 9 | LOW |
| Sharma N,2016 | Status and determinants of birth preparedness and complication readiness in a rural block of Haryana | Yes | Yes | Yes | Yes | Yes | unclear | Yes | Yes | Yes | 8 | LOW |
| Kusuma YS,2018 | Birth preparedness and determinants of birth place among migrants living in slums and slum-like pockets in Delhi, India | Yes | Yes | Unclear | Yes | Yes | Yes | Yes | Yes | No | 7 | LOW |
| Viswanathan VT,2020 | Study to Assess Birth Preparedness and Complication Readiness to Promote Safe Motherhood among Women from a Rural Area of Western Maharashtra | Yes | Yes | Yes | Unclear | Yes | Yes | Yes | Yes | Yes | 8 | LOW |
| Acharya AS ,2015 | Making Pregnancy Safer-Birth Preparedness and Complication Readiness Study Among Antenatal Women Attendees of A Primary Health Center, Delhi | Yes | No | Unclear | Yes | Yes | Yes | Yes | Yes | No | 6 | MEDIUM |
| Agarwal S,2010 | Birth Preparedness and Complication Readiness among Slum Women in Indore City, India | Yes | Yes | Yes | Yes | Yes | Yes | Yes | Yes | Yes | 9 | LOW |
| Gurung J,2017 | Birth Preparedness And Complication Readiness Among Rural Pregnant Women: A Cross-Sectional Study In Udupi, Southern India | Yes | Yes | Yes | Yes | Yes | Yes | Yes | Yes | Yes | 9 | LOW |
| Gupta S,2016 | Birth Preparedness And Complication Readiness Plans Among Antenatal Attendees At Primary Health Centre Of District Jhansi, Up, India | Unclear | No | Unclear | Yes | Unclear | Yes | Yes | Yes | No | 4 | MEDIUM |
| Kar M,2019 | Birth Preparedness And Complication Readiness Among Pregnant And Recently Delivered Women In Villages Of A Block Of Ganjam, Odisha, India: A Community Based Cross-Sectional Study | Yes | Yes | Yes | Yes | Yes | Yes | Yes | Yes | Yes | 9 | LOW |
| Karir S,2022 | Birth Preparedness And Complication Readiness Among Recently Delivered Mothers-A Cross Sectional Survey In An Urban Community In Eastern India | Yes | Yes | Yes | Yes | Yes | Yes | Yes | Yes | Yes | 9 | LOW |
| Rajesh P,2016 | A Study To Assess The Birth Preparedness And Complication Readiness Among Antenatal Women Attending District Hospital In Tumkur, Karnataka, India | Yes | Yes | unclear | Yes | Yes | Yes | Yes | Yes | No | 7 | LOW |
| Rakesh J,2017 | Birth Preparedness And Complication Readiness Among Women Availing Obstetric Services At A Rural Maternity Hospital In South Karnataka, India | Yes | No | Unclear | Yes | Yes | Yes | Yes | Yes | No | 6 | MEDIUM |
| Bhilwar M, 2021 | Birth Preparedness And Complication Readiness Among Pregnant Women Visiting A Government Hospital In Delhi, India | Yes | Yes | Yes | Yes | Yes | Yes | Yes | Yes | Yes | 9 | LOW |
| Shastri VD,2019 | Birth Preparedness For Safe Delivery, Readiness Planning And Associated Factors Among Mothers In North India: A Cross-Sectional Study In Bihar, India | Yes | Yes | unclear | Yes | unclear | Yes | Unclear | Yes | No | 5 | MEDIUM |
| Chajhlana SPS,2018 | Status Of Birth Preparedness And Complication Readiness Among Pregnant Women In Rural Areas | Unclear | No | Unclear | Yes | Yes | Yes | Yes | Yes | No | 5 | MEDIUM |
| Chandrakar T,2022 | Evaluation Of Birth Preparedness And Complication Readiness Index Among Women Of Central India: A Community-Based Survey Of Slums | Yes | Yes | Yes | Yes | Yes | Yes | Yes | Yes | Yes | 9 | LOW |
| Sulekha T,2020 | Knowledge And Practice Of Birth Preparedness And Complication Readiness Among Rural Mothers: A Cross-Sectional Study On 17 Villages Of South Karnataka | Yes | Yes | Yes | Yes | Yes | Yes | Yes | Yes | No | 8 | LOW |
| Salroo F,2022 | Birth Preparedness And Complication Readiness Among Pregnant Women Attending A Maternal And Child Care Hospital Of Government Medical College In South Kashmir, India: Across-Sectional Study | Yes | No | Yes | Yes | Yes | Yes | Yes | Yes | Yes | 8 | LOW |
| Ghosh A,2017 | Status Of Birth Preparedness And Complication Readinessamong Recently Delivered Women:A Community Based Study In A Slum Of Kolkata, West Bengal | Yes | No | Yes | Yes | Yes | Yes | Yes | Yes | Yes | 8 | LOW |
| Sau B, 2021 | Assessment Of Birth Preparedness And Complication Readiness Among Postnatal Mothers In Tertiary Care Hospital, West Bengal | Yes | No | Yes | Yes | Yes | Yes | Yes | Yes | Yes | 8 | LOW |
| Patel G,2022 | Birth Preparedness And Complication Readiness Among Pregnant Women Attending An Urban Health Centre In Surat, India | Yes | No | Unclear | Yes | Yes | Yes | Yes | Yes | Yes | 7 | LOW |
| Mutreja S,2015 | Knowledge And Practice Of Birth Preparedness Among Tribal Women In Sukma District Of Chhattisgarh, India | Yes | Yes | Unclear | Yes | Yes | Yes | Yes | Yes | No | 7 | LOW |
| Indira NC,2021 | Birth Preparedness And Complication Readiness Among Antenatal Mothers Attending Tertiary Care Hospital | Yes | Yes | No | Yes | Yes | Yes | Yes | Yes | No | 7 | LOW |
| Patil AA,2022 | Level Of Birth Preparedness And Complication Readiness Among Pregnant Women Residing In Urban Slums Of Shivamogga City, India | Yes | Yes | Yes | Yes | Yes | Yes | Yes | Yes | Yes | 9 | LOW |
| Patel NA,2017 | Birth preparedness: studying its effectiveness in improving maternal health in urban slums of Jamnagar, Gujarat | Yes | Yes | Yes | Yes | Unclear | Yes | Unclear | Yes | Yes | 7 | LOW |
| Patil MS,2016 | Birth preparedness and complication readiness among primigravida women attending tertiary care hospital in a rural area | Yes | No | Yes | Yes | Yes | Yes | Yes | Yes | Yes | 8 | LOW |
| Waghmare R,2018 | Status of birth preparedness and complication readiness among pregnant women in field practice area of rural health and training centre, manglia, indore | Yes | Unclear | Unclear | Yes | Unclear | Yes | Unclear | Yes | No | 4 | MEDIUM |
| VidhyaShree MD,2020 | Birth preparedness and complication readiness for a safe motherhood among antenatal women attending an urban health centre, Pudupet | Yes | Yes | Yes | Yes | Yes | Yes | Yes | Yes | Yes | 9 | LOW |
| Mazumdar R,2014 | Status of birth preparedness and complication readiness in a rural community: a study from West Bengal, India | Yes | Yes | Yes | Yes | Yes | Yes | Yes | Yes | Yes | 9 | LOW |
